# Prevalence, Virulence Genes, Antimicrobial Susceptibility, and Genetic Diversity of *Staphylococcus aureus* from Retail Aquatic Products in China

**DOI:** 10.3389/fmicb.2017.00714

**Published:** 2017-04-20

**Authors:** Dongli Rong, Qingping Wu, Mingfang Xu, Jumei Zhang, Shubo Yu

**Affiliations:** ^1^Guangdong Institute of Microbiology, State Key Laboratory of Applied Microbiology Southern China and Guangdong Provincial Key Laboratory of Microbial Culture Collection and Application, Guangdong Open Laboratory of Applied MicrobiologyGuangzhou, China; ^2^College of Life Science and Technology, Jinan UniversityGuangzhou, China

**Keywords:** *Staphylococcus aureus*, aquatic products, antibiotic resistance, virulence genes, multilocus sequence typing, staphylococcal cassette chromosome *mec*

## Abstract

*Staphylococcus aureus* is an important food-borne opportunistic pathogen that frequently causes severe blood and tissue infections or even fatal illnesses. Although *S. aureus* has been extensively studied in livestock and poultry foods in China, limited information has been reported in aquatic products. Accordingly, in this study, we aimed to characterize *S. aureus* in aquatic products purchased from retail markets in China. In total, 320 aquatic food samples were collected from 32 provincial capitals in China. The results showed that 119 samples (37.2%, 119/320) were positive for *S. aureus* by both qualitative and quantitative analyses. The contamination levels of 78.2% of samples ranged from 0.3 to 10 MPN/g, and six samples exceeded 110 MPN/g. A total of 119 *S. aureus* isolates from positive samples were selected to evaluate virulence factors, antibiotic resistance, and molecular characteristics. All *S. aureus* isolates were evaluated for the presence of 11 virulence genes by multiplex polymerase chain reaction, and α-hemolysin (*hlα*, 84.9%), fibronectin-binding protein A (*fnbA*, 79.0%), *S. aureus* enterotoxin E (*see*, 53.8%), and Panton-Valentine leucocidin (*pvl*, 50.4%) were identified as the major genes. These genes formed 56 different profiles, with the major profile identified as *pvl*-*hlα*-*fnbA* (28.6%). The antimicrobial susceptibility of all isolates was analyzed through the disk diffusion method, and the results showed high resistance to β-lactams, macrolides and tetracyclines, but susceptibility to linezolid and vancomycin. In addition, 26 sequence types (STs) were obtained via multilocus sequence typing, including seven novel STs, among which ST1 (20.2%), ST15 (18.5%), and ST188 (13.4%) were the most common STs. All the isolates were *mecC* negative, but nine isolates carrying *mecA* were evaluated by staphylococcal cassette chromosome *mec* (SCC*mec*) typing, all of which were SCC*mec*III or SCC*mec*IV types. Isolates of *SCCmec*III showed a high prevalence and were multidrug resistant. Our results showed that aquatic products could be a vehicle for transmission of virulence genes and multidrug-resistant *S. aureus*, representing a potential public health risk. The STs identified in this study indicated the genetic diversity of *S. aureus*, thereby providing important basic data for the dissemination of *S. aureus* in aquatic products.

## Introduction

*Staphylococcus aureus* is an important opportunistic pathogen widely presented in the natural environment. About 20–30% of healthy people harbors the microorganism in the nares and about 60% of the population intermittently harbors (Kluytmans et al., 1997). The elderly, infants, and immunocompromised patients are highly susceptible to *S. aureus* infection (Murray, 2005; Foster et al., 2014). Additionally, *S. aureus* not only causes many diseases, including abscesses, endocarditis, sepsis, and necrotizing pneumonia, but also transmits between humans and animals (Lowy, 1998; David and Daum, 2010). This pathogen can spread from production equipment and food handlers to the food chain (Sospedra et al., 2012). Moreover, methicillin-resistant *S.aureus* (MRSA) infection causes morbidity and mortality, resulting in serious economic burden (Klevens et al., 2007; Centers for Disease Control and Prevention, 2013).

Despite extensive research efforts, many individuals still suffer from diseases caused by *S. aureus* (Lee et al., 2013). The pathogenicity of *S. aureus* is related to various virulence factors, including *S.aureus* enterotoxins (SEs), fibronectin-binding proteins A and B (FnBPA and FnBPB), Panton-Valentine leucocidin (PVL), hemolysins (Hlα and Hlβ), and toxic shock syndrome toxin-1 (TSST-1), most of which are involved in the adherence, colonization, and tissue invasion abilities of the pathogen, thereby promoting pathogenicity (Puah et al., 2016). Currently, 23 SEs and SE-like toxins (SEls) have been identified; these proteins are resistant to proteolysis and are heat-stable (Sato et al., 2014). Ingesting from 20 ng to 1 μg SEs can cause symptoms in humans (Normanno et al., 2007). The mechanisms of SEs causing food poisoning are not clear. However, SEs in Staphylococcal food-borne diseases (SFDs) was verified that it directly affects the intestinal epithelium and vagus nerve, causing stimulation of the emetic center (Hennekinne et al., 2012). The classic enterotoxins A-E have been frequently isolated from outbreaks of food poisoning in more than 90% of cases; among these cases, SEA was found to be the most common cause of staphylococcal food poisoning worldwide, and SEE has been implicated in the SFD outbreaks in USA, UK, and France (Bergdoll et al., 1971; Angeles Argudin et al., 2010). Moreover, owing to the remarkable toxicity and stability of *S. aureus*, this organism is considered a potential biochemical weapon (King et al., 1999).

In recent years, global access to effective antimicrobials has become a major concern, and over 700,000 deaths worldwide, including 214,000 neonatal sepsis deaths, have been reported owing to the increase in multidrug resistance pathogen strains (Laxminarayan et al., 2016; O’Neill, 2016).

*Staphylococcus aureus* is a major pathogen causing nosocomial- and community-associated infections and has become a major problem owing to the prevalence of antibiotic resistance in this organism, particularly MRSA strains. The *mecA-* or *mecC-* positive were considered MRSA strains. Currently, *mecC* MRSA strains have been found from 14 different host species (Paterson et al., 2014), but no *mecC-* positive were found from the aquatic products sample. The study from Sweden indicated that the *mecC* was poor colonizer and resulted in few secondary disease cases (Lindgren et al., 2016). Staphylococcal cassette chromosome *mec* (SCC*mec*) typing is a suitable method for detection of MRSA strains, allowing acquisition of different SCC*mec* types. Eleven different SCC*mec* types have been reported, among these, SCC*mec* I, II, and III are known as predominant hospital-acquired types, and community MRSA isolates are usually SCC*mec* IV or V (Lim et al., 2012). To gain insights into the relatedness of strains, potential sources of infection, routes of transmission, and presence of virulence and resistance, genotypic and phenotypic methods can be employed (Kim et al., 2014). Multilocus sequence typing (MLST) provides an excellent tool for investigating the population structure of *S. aureus* globally and can be used to determine the potential spread of pathogenic genes to other bacterial species by identifying whether the genes are located on mobile genetic elements (Saunders and Holmes, 2007). Although MRSA are more significantly associated with morbidity than methicillin-susceptible *S. aureus* (MSSA), both strains are major causes of infections in nosocomial and communities.

*Staphylococcus aureus* can contaminate various foods, such as meats, poultry, fish, eggs, dairy products, and salads (Hennekinne et al., 2012). Of these, aquatic products provide an average of one-fifth of the total animal protein intake for the world population (Cajascano, 2014). *Vibrio parahaemolyticus, Salmonella*, and *Listeria monocytogenes* have been reported as microbiological hazards of aquatic products in China (Xu et al., 2014; Wu et al., 2015; Yang et al., 2015). However, few studies have evaluated the prevalence of *S. aureus* in aquatic products in China. Some studies in Japan, Spain, and South Korea have reported the contents of *S. aureus* in aquatic products (Hammad et al., 2012; Vazquez-Sanchez et al., 2012; Cho et al., 2014), all these reports have suggested that *S. aureus* in aquatic products may represent a potential risk to consumers. Moreover, food poisoning causing by consumption of *S. aureus* contaminated fish has been reported both in the USA and Hong Kong (Iwamoto et al., 2010). Eating raw fish has become popular owing to its taste, novelty value, and nutritional benefits, which added to the threat of food poisoning due to *S. aureus* contaminated aquatic products.

Therefore, in this study, we determined the prevalence, virulence factors, antibiotic resistance, and molecular genetics of *S. aureus* in aquatic products in China, providing an important basic data for the risk assessment of *S. aureus* in aquatic food.

## Materials and Methods

### Sampling

**FIGURE 1:**
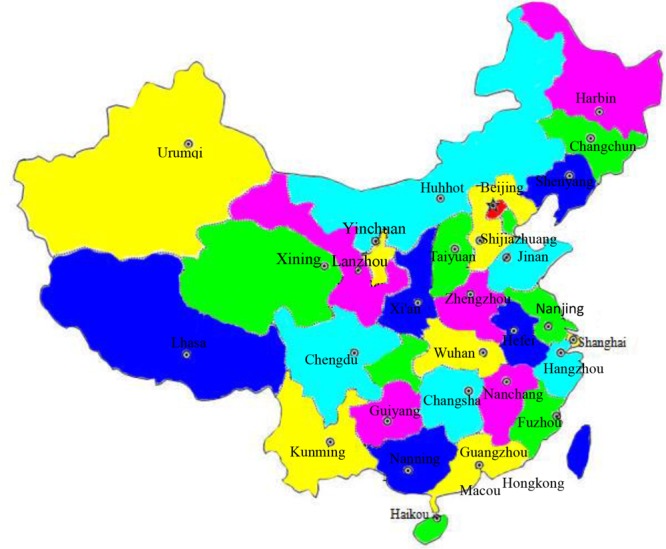
**Map of China showing the locations of the 32 metropolitan cities where the *Staphylococcus aureus* strains were collected**.

A total of 320 raw aquatic food samples were collected from 64 retail outlets and 64 commercial hypermarkets, in 32 provincial capitals of China, from June 2015 to June 2016 (**Figure 1**). Samples included freshwater fish (*n* = 142), saltwater fish (*n* = 113), and shrimp (*n* = 65). All of the samples were stored tightly inside sealed aseptic bags, surrounded by a biological ice bag, and placed in a box maintained at a temperature lower than 4°C. Samples were immediately transported to the laboratory and subjected to microbiological analysis within 24 h.

### Qualitative and Quantitative Detection of *S. aureus*

All of the samples were subjected to qualitative and quantitative analysis for *S. aureus* using an nrichment method described by the National Food Safety Standard of China-Food microbiological examination, *S.aureus* (GB 4789.10-2010), with slight modifications for qualitative detection. The most probable number (MPN) method was included in quantitative analysis by applying nine-tube MPN method, and the values were determined based on the number of positive tube(s) in each of the three sets and the MPN table. Briefly, 25.0 g sample was randomly collected from each aquatic food product and was pre-enriched in 225 mL tryptic soy broth with 10% sodium chloride (Huankai Microbial Sci & Tech, Co., Ltd, Guangzhou, China). After homogenization, the sample were divided into three groups of a concentration gradient, with three tubes per gradient, representing 1.0, 0.1, and 0.01 g of the original sample, respectively. Then the tubes were incubated at 36 ± 1°C for 48 h. A loopful of the broth enrichment culture was streaked onto *S. aureus-*selective chromagar plates (Guangdong Huankai Microbial Sci & Tech, Co., Ltd, Guangzhou, China) and incubated at 36 ± 1°C for 24 h. One or more typical single colonies were selected for Gram staining, after purification for 24 h at 36 ± 1°C on *S. aureus*-selective chromagar plates. One or more colonies were selected and incubated at 36 ± 1°C for 24 h in brain heart infusion broth (BHI), and 1 mL bacterium solution was then subjected to coagulase tests, incubated 36 ± 1°C, and observed every 30 min for 6 h. The results were judged as positive when the solidification or solidification volume was greater than half the original volume.

Confirmation of *S. aureus* was performed using API STAPH identification test strips (BioMerieux, Marcy-l, Etoile, France) according to the manufacturer’s instructions.

### Detection of Virulence Genes

The DNA of *S.aureus* isolates were extracted using the Genomic DNA Extraction kit (Majorbio, China) according to the manufacturer’s instructions. The concentration of DNA was assessed using a spectrophotometer. Eleven virulence genes were detected by multiple-PCR using appropriate specific primers (Jarraud et al., 2002; Tristan et al., 2003). All primers were chemically synthesized by BGI Biotech, Co., Ltd (Beijing, China). The PCR mixture (25 μL) contained 0.6 μM primers, 50 ng DNA template, 12.5 μL master mix, and sterile purified water (to 25 μL). PCR conditions included initial denaturation (94°C for 4 min), followed by 30 cycles of denaturation (94°C for 45 s), annealing (55°C for 30 s), and extension (72°C for 45 s), and a final extension (72°C for 8 min).

### Antimicrobial Susceptibility Testing

Antimicrobial susceptibility was evaluated using the Kirbye-Bauer disk diffusion method in accordance with The Clinical and Laboratory Standards Institute [CLSI] (2015), the following 24 antimicrobial agents were tested: penicllin G (P, 10 U), ampicillin (AMP, 10 μg), amoxicillin- clavulanic acid (AMC, 30 μg), cefoxitin (FOX, 30 μg), ceftazidime (CAZ, 30 μg), cefepime (FEP, 30 μg), amikacin (AK, 30 μg), gentamicin (CN, 10 μg), kanamycin (K, 30 μg), streptomycin (S, 10 μg), quinupristin/dalfopristin (QD, 15 μg), norfloxacin (NOR, 10 μg), ciprofloxacin (CIP, 5μg), erythromycin (E, 15 μg), telithromycin (TEL, 15 μg), chloramphenicol (C, 30 μg), tetracycline (TE, 30 μg), trimethoprim-sulfamethoxazole (SXT, 25 μg), linezolid (LZD, 30 μg), vancomycin (VA, 30 μg), fusidic acid (FD, 10 μg), rifampicin (RD, 5 μg), fosfomycin (FOS, 200 μg), and clindamycin (DA, 2 μg). The isolates were also examined using a microdilution test to determine minimum inhibitory concentrations (MICs) according to the CLSI method for vancomycin MICs. In addition, the MRSA strains were identified by cefoxitin disk diffusion method (The Clinical and Laboratory Standards Institute [CLSI], 2015).

### MLST and SCC*mec*-Typing

Typing methods were carried out based on sequencing of seven conserved housekeeping genes (i.e., *arcC, aroE, glpF, gmk, pta, tpi*, and *yqiL*) representing the stable “core” of the staphylococcal genome. Each gene fragment was translated into a distinct allele, and each isolate was classified as a sequence type (ST) by the combination of alleles of the seven housekeeping loci^1^. MLST was used to valuated evolutionary relationships of all isolates, and PCR was performed to obtain *mecA*- or *mecC*- positive isolates. Then the SCC*mec* type of the positive isolates were determined using a multiplex PCR method, as previously described (Zhang et al., 2005). MRSA isolates with unanticipated fragments or lacking fragments by multiplex PCR were defined as non-typeable (NT).

## Results

### Prevalence and Quantitative Analysis

We collected and tested a total of 320 aquatic products samples from 32 provincial capitals in China. One hundred and nineteen (37.2%, 119/320) samples were positive for *S. aureus* by both qualitative and quantitative analyses; one strain from each sample was selected for subsequent analyses. The contamination of *S.aureus* was most common among freshwater fish samples (74/142; 52.1%), followed by shrimp (18/65; 27.7%), and saltwater fish (27/11; 23.9%). Based on quantitative analysis, 78.2% (93/119) of positive samples were contaminated at levels ranging from 0.3 to 10 MPN/g, and 16.8% (20/119) of samples were contaminated at levels ranging from 10 to 110 MPN/g, with six positive samples from Haikou, Changsha, Chengdu, Hangzhou, Jinan, and Yinchuan cities showing contamination levels exceeding 110 MPN/g (**Table 1**).

**Table 1:** Prevalence of *Staphylococcus aureus* in retail aquatic products in China.

Species of fish	No. of samples	No. of positive samples	Positive rate	No. of positive samples by quantitative methods (MPN/g)			
				0.3–1	1–10	10–110	>110
Freshwater fish	142	74	52.1%	25	29	15	5
Saltwater fish	113	27	23.9%	18	6	2	1
Shrimp	65	18	27.7%	10	5	3	0
Total	320	119	37.2%	53	40	20	6

### Prevalence of Virulence Genes

Over 119 *S. aureus* isolates were evaluated for the presence of 11 virulence genes, and 56 different profiles were observed, the major profiles were *pvl*-*hlα*-*fnbA* (28.6%), *see*-*hlα*-*fnbA* (19.6%), and *sea*-*see*-*pvl*-*hlα*-*fnbA* (17.9%). The major virulence genes were present in the following order (from most to least prevalent): *hlα* (101/119, 84.9%), *fnbA* (94/119, 79.0%), *see* (64/119, 53.8%), and *pvl* (76/119, 63.9%); however, only three strains of bacteria carried *tsst-1* (**Table 2** and Supplementary Table 1).

**Table 2:** Virulence genes of *S. aureus* in retail aquatic products in China.

Sources	Positive samples	*sea*	*seb*	*sec*	*sed*	*see*	*pvl*	*hlα*	*hlβ*	*fnbA*	*fnbB*	*tsst-1*
Freshwater fish	74	15	7	25	8	38	41	62	4	57	12	3
Saltwater fish	27	9	4	9	5	13	15	23	2	21	3	0
Shrimp	18	3	1	8	3	13	4	16	2	16	3	0
Total	119	27	12	42	16	64	60	101	8	94	18	3
Percentage (%)		22.7	10.1	35.3	13.4	53.8	50.4	84.9	6.7	79.0	15.1	2.5

### Antimicrobial Susceptibility of the *S. aureus* Isolates

All *S. aureus* isolates were evaluated for antimicrobial susceptibility to 24 antibiotics. A total of 105 (88.2%) *S. aureus* isolates were resistant to ampicillin and penicillin G, and 88 (73.9%) were resistant to amoxicillin-clavulanic acid. The predominant multidrug resistant profiles among the isolates were evaluated. 90.6% of isolates displayed resistant to three or more agents, and 30.3% of isolates displayed resistant to six or more agents, among which 5.9% of isolates displayed resistant to nine or more agents. All *S. aureus* isolates were susceptible to linezolid and vancomycin. Ten isolates were verified as MRSA by cefoxitin disk diffusion tests, although only nine isolates were *mecA* positive, as determined by PCR. Some of the MRSA clones contained isolates in which the *mec* genes were lost or inactivated (Enright et al., 2000). Almost all of the MRSA isolates displayed resistant to six β-lactam antibiotics (penicillin, ampicillin, amoxicillin- clavulanic acid, ceftazidime, cefepime, and cefoxitin), and the highest level of resistance (showing resistant to 15 antibiotics) was observed in sample 2704 from Shanghai. The antimicrobial resistance profiles of the *S. aureus* strains are shown in **Table 3** and Supplementary Table 1.

**Table 3:** Results of antimicrobial resistance of *S. aureus* isolates in the study.

Category	Antimicrobial agents	*S. aureus* (*n* = 119)		
		Resistant (R)	Intermediate (I)	Susceptible (S)
**I**	**β-Lactams**			
	Ampicillin (10 μg)	105 (88.2%)	0 (0.0%)	14 (11.8%)
	Penicllin G (10 units)	105 (88.2%)	0 (0.0%)	14 (11.8%)
	Amoxicillin-clavulanic acid (30 μg)	88 (73.9%)	0 (0.0%)	31 (26.1%)
	Cefoxitin (30 μg)	10 (8.4%)	0 (0.0%)	109 (91.6%)
	Ceftazidime (30 μg)	13 (10.9%)	0 (0.0%)	106 (89.1%)
	Cefepime (30 μg)	10 (8.4%)	0 (0.0%)	109 (91.6%)
**II**	**Aminoglycosides**			
	Kanamycin (30 μg)	27 (22.7%)	7 (5.9%)	85 (71.4%)
	Streptomycin (10 μg)	17 (14.3%)	6 (5.0%)	96 (80.7%)
	Amikacin (30 μg)	2 (1.7%)	0 (0.0%)	117 (98.3%)
	Gentamicin (10 μg)	5 (4.2%)	1 (0.8%)	113 (95.0%)
**III**	**Quinolones and fluoroquinolones**			
	Norfloxacin (10 μg)	8 (6.7%)	8 (6.7%)	103 (86.6%)
	Ciprofloxacin(5 μg)	6 (5.0%)	3 (2.5%)	110 (92.5%)
	Quinupristin/dalfopristin (15 μg)	3 (2.5%)	0 (0.0%)	116 (97.5%)
**IV**	**Macrolides**			
	Erythromycin (15 μg)	64 (53.8%)	5 (4.2%)	50 (42.0%)
	Telithromycin (15 μg)	10 (8.4%)	4 (3.4%)	105 (88.2%)
**V**	**Tetracyclines**			
	Tetracycline (30 μg)	36 (26.9%)	1 (0.8%)	82 (68.9%)
**VI**	**Lincosamides**			
	Clindamycin (2 μg)	15 (12.6%)	3 (2.5%)	101 (84.9%)
**VII**	**Phenicols**			
	Chloramphenicol (30 μg)	9 (7.5%)	1 (0.8%)	109 (91.6%)
**VIII**	**Sulfonamides and synergistic agents**			
	Trimethoprim-sulfamethoxazole (25 μg)	9 (7.5%)	0 (0.0%)	110 (92.5%)
**IX**	**Oxazolidinones**			
	Linezolid (30 μg)	0 (0.0%)	0 (0.0%)	119 (100.0%)
**X**	**Glycopeptides**			
	Vancomycin (MIC)	0 (0.0%)	0 (0.0%)	119 (100.0%)
				
**The others**	Rifampicin (5 μg)	3 (2.5%)	0 (0.0%)	116 (97.5%)
	Fusidic acid (10 μg)	4 (3.4%)	0 (0.0%)	115 (96.6%)
	Fosfomycin (200 μg)	1 (0.8%)	7 (5.9%)	111 (93.3%)
**Pansusceptible**				
	≥3 Antimicrobia	108 (90.6%)		
	≥6 Antimicrobia	39 (30.3%)		
	≥9 Antimicrobia	7 (5.9%)		

*The font bold represents the antimicrobial category.*

### MLST and SCC*mec-*Typing

**FIGURE 2:**
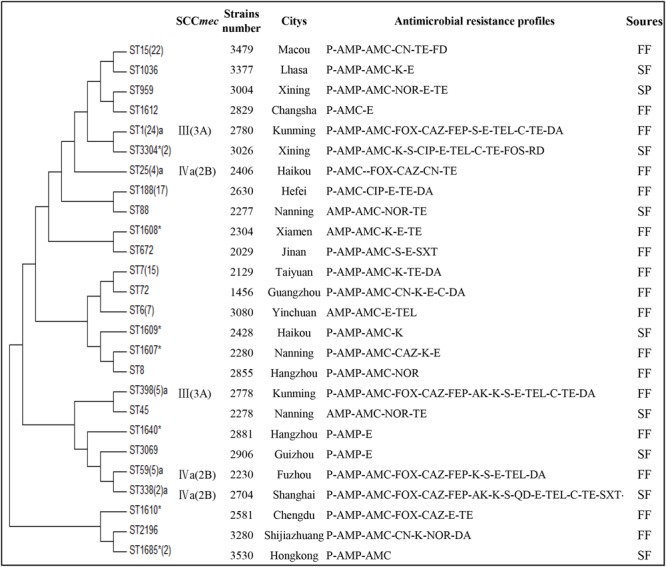
**Multilocus sequence typing (MLST) minimum evolution tree and multidrug resistance of *S. aureus* in aquatic food products isolates**. The tree was built with Mega seven software using concatenated sequences. FF, freshwater fish; SF, saltwater fish; SP, shrimp; ^∗^ indicates the novel STs; ^a^indicates MRSA isolates. The isolates of ST1685 were found two novel loci (aro: 207, ypi-L: 488).

All *S. aureus* isolates were analyzed by MLST using the sequences generated from internal fragments of seven housekeeping genes. Overall, 26 STs were obtained; the most common STs were ST1 (20.2%), ST15 (18.5%), and ST188 (13.4%); seven new STs (ST1607, ST1608, ST1609, ST1610, ST1612, ST1685, and ST3304) and two novel loci (aro: 207, ypi-L: 488) were found. Nine isolates were *mecA-* positive, and all the isolates were *mecC-* negative. The *mecA*-positive isolates were mainly SCC*mec*III and SCC*mec*IV, and the STs including ST59, ST338, ST398, ST1, and ST25 (**Figure 2** and Supplementary Table 1).

## Discussion

Data on the microbial safety of *S. aureus* in aquatic products in China are limited. In this study, *S. aureus* was detected in 37.2% of retail aquatic products samples, suggesting a similar prevalence to previous studies of fishery products in Northwest Spain (43.0%) (Vazquez-Sanchez et al., 2012) and Korea (40.7%) (Cho et al., 2014), but lower than the report for raw fish from Japan (87.0%) (Hammad et al., 2012). However, the prevalence of in this study was higher than that in samples of raw chicken (24.2%) in China (Wang et al., 2013) and samples from fresh meat (28.1%) in Shanghai, China (Song et al., 2015). In our study, freshwater fish samples (52.1%) showed higher *S. aureus* prevalence and microbial loads than saltwater fish samples (23.9%), and five freshwater fish samples exceeded 110 MPN/g. Additionally, nearly 80.0% of MRSA isolates were obtained from freshwater fish, which highlighted the threat of *S. aureus* in freshwater fish contributed to the higher antibiotic resistance of MRSA isolates. The major reason for higher prevalence of *S. aureus* in freshwater fish samples is probably resulted from pollution by human or animal feces and sewage in freshwater compared to pollution-free saltwater (Yang et al., 2015; Gomez et al., 2016; Boopathy, 2017). The prevalence and enumeration data of this national-wide systematic survey demonstrated a substantial risk to public health and suggests that Chinese food safety management should further strengthen surveillance of aquatic products.

Staphylococcal food poisoning, a frequent cause of food-borne gastroenteritis worldwide, results from ingestion of one or more SEs produced by *S. aureus* in foods (Angeles Argudin et al., 2010). SEs are heat-stable proteins and major causes of food poisoning (Hennekinne et al., 2012). Consumption of less than 1 μg of SEs can lead to nausea, vomiting, abdominal pain, cramps, and diarrhea (Pinchuk et al., 2010). In this study, the classical SEs (*sea*-*see*) were detected. The incidence of *see* was 53.8%, which was higher than that in a previous study in Iran (18.4%) (Mashouf et al., 2015). The incidences of the other SEs were lower and similar to those reported in Italy and Iran (Normanno et al., 2005; Mashouf et al., 2015). The involvement of *see* in food poisoning has rarely been reported in China. However, three cases of food poisoning caused by SEE were reported in USA, France (Bergdoll et al., 1971; Morris et al., 1972) and Shaanxi, China (Li et al., 2015). Similarly, Ostyn et al. (2010) reported that 90 ng of SEE can lead to food poisoning. Poor sanitation and hygiene in communities and hospitals facilitate the spread of virulence factors and increase the frequency of global, travel, trade, and disease transmission (Laxminarayan et al., 2013). Therefore, it is necessary to improve sanitation conditions in order to ensure food safety.

The pathogenicity of *S. aureus* is a complex process, which involved in mediating adhesion to and invasion of different types of hosts as well as escaping of immune responses via coordinate expression during different stages of infection (Yang and Ji, 2014). The 119 *S. aureus* isolates in this study were screened for 11 virulence genes to gain insights into their potential pathogenic ability; the *fnbA*-*hlα*-*pvl* gene profile was detected at high levels. Fibronectin-binding proteins have been shown to have important roles in the establishment of infections and persistent infections; *α*-hemolysin leads to disruption of the epithelial barrier and homeostasis, which in turn lead to necrotic cell death and systemic infection (Kong et al., 2016); and PVL is a pore-forming cytotoxin. Notably, the *hlα* gene was reported to be responsible for an *S. aureus* food poisoning outbreak in Xi’an, China (Li et al., 2015). Indeed, approximately 90.0% of *S. aureus* induced necrotizing pneumonia is associated with isolation of *S. aureus* strains carrying the *pvl* gene (Bhatta et al., 2016). A previous report described the presence of PVL in aquatic products, but found that no samples of ready-to-eat raw fish were positive for PVL in Japan (Hammad et al., 2012). In contrast, our findings showed that 60 isolates (50.4%) contained PVL. This result demonstrated the potential hazards of *S. aureus* for food handlers and consumers owing to the high levels of toxicity.

*Staphylococcus aureus* is known not only for its virulence factors but also for its multidrug resistance. Additionally, this bacterium can acquire resistance in more than one way, including natural spontaneous mutations, induced mutations, transduction, conjugation, and transformation (Skovgaard, 2002). In order to increase production in aquaculture, farmers use different antibiotics to prevent and treat pathogenic bacterial infections in aquatic animals (Cabello et al., 2013; Huang et al., 2015). The total production of all antibiotics in China was estimated to be 248,000 tons for 2013, in which 53,800 tons of antibiotics entered into the receiving environment following various wastewater treatments (Zhang et al., 2015). Indeed, many bacteria come in close contact with many types of antibiotics discharged in wastewater, these processes contribute to resistance by overexposing cultures to these bactericidal or bacteriostatic chemicals (Everage et al., 2014). This may lead to development of antibiotic-resistant bacteria. In our study, 90.6% of isolates displayed three or more antimicrobial- resistant profiles, among which 30.3% of isolates displayed resistance to six or more antimicrobial-resistant profiles; this was higher than that observed in ready-to-eat foods in China (Yang et al., 2016). We found that both MRSA and MSSA were multidrug resistance and showed high levels of resistant to ampicillin, penicillin G, amoxicillin-clavulanic acid, streptomycin, erythromycin, kanamycin, tetracycline and clindamycin, all of which are still widely used in human therapy in China because of their low cost and availability (Zhou et al., 2013; Zhang et al., 2016). These results suggested that the corresponding risk prevention and control measures were urgently demanded to reduce emerging antimicrobial-resistant strains. Fortunately, no vancomycin- intermediate *S. aureus* or vancomycin-resistant *S. aureus* isolates were found. In addition, the Chinese government launched a national action plan to curb bacterial resistance in 2016^2^, highlighting the necessity for robust management and monitoring of antibiotic use.

Currently, many epidemic clones are in circulation in Asia, and few data were available, particularly for *S. aureus* genotypes in aquatic products, making it difficult to determine whether strains were from human or aquatic origin. MLST revealed different genetic characteristics in the varying geographic regions. ST1, which was most prevalent in this study, is popular in the USA and Europe (Chuang and Huang, 2013). The second most common clone in this study was ST15, which has been previously reported among animals and humans and is common to regional communities and hospitals (Jamrozy et al., 2012). ST188, one of the most prevalent clone in this study, has also been reported to be associated with SFP Shenzhen and Hong Kong in China (Yan et al., 2012; Song et al., 2016).

Studies have shown that ST clones of *S. aureus* often display different antibiotic resistance patterns (Goudarzi et al., 2016). High antimicrobial resistance could also be observed in MSSA strains belonging to ST7 (average resistance to seven types of antibiotics), with MSSA ST188, ST1, and ST15 (average resistance to five types of antibiotics) displaying a lower prevalence of resistance determinants in this study. A previous study reported the ST7 type of *S. aureus* isolated from patients with skin and soft tissue infections (SSTIs) in China (Yu et al., 2015); in contrast, ST7 was the predominant molecular type of MRSA strain in a sample from Guangzhou, China (Fan et al., 2016).

The resistance of MRSA isolates is generally high, in our study nine MRSA isolates were *mecA-*positive but *mecC-*negative. Yang et al. (2016) have shown that no *mecC*-positive isolates were identified from 550 ready-to-eat samples from China. Two MRSA lineages were identified in aquatic products from China in this study: CA-MRSA-III (ST59, ST338, ST398, and ST1) and CA-MRSA -IVa (ST25, ST59, and ST338). A previous study has showed that isolates of nosocomial SCCmec IV and SCCmec III spread from the hospital to the community and vice versa in China (Dan et al., 2010). In this study, 33.3% of MRSA isolates were ST59, consistent with the report that ST59 was the most common clone among MRSA isolates in China (Yu et al., 2015; Yang et al., 2016). Moreover, all of these MRSA isolates harbored the *pvl*-*hlα*-*fnbA* profile, which may cause SSTIs, necrotizing pneumonia, and sepsis. In addition, our results showed that isolates harboring MRSA-III were more resistant to non-β-lactam antibiotics (e.g., chloramphenicol, streptomycin, and telithromycin) than the isolates harboring type MRSA-IVa. Thus, MRSA-III may be a major threat to public health.

In this study, we identified seven new STs: ST1607, ST1608, ST1609, ST1610, ST1612, ST1685, and ST3304. Most of these STs displayed a relatively lower prevalence of resistance determinants, except ST3304 isolates, which had only one variable locus relative to that of ST1. However, the number of resistant ST3304 isolates was significantly higher than the number of resistant ST1 isolates, suggesting that the isolates may have obtained resistance during evolution. At present, there are nearly 4,000 STs of *S.aureus* globally^1^, and this number continues to increase. New STs may have the inherent advantage of carrying multidrug-resistant or virulent factors. Thus, further studies are needed to elucidate the evolutionary mechanisms mediating drug resistance and virulence in order to prevent greater threats to human health.

## Conclusion

This is the first comprehensive report of the prevalence of *S. aureus* virulence factors, antibiotic resistance phenotypes, and molecular typing in retail aquatic food products from diverse regions of China. Our results showed that there was a high incidence of *S.aureus* and that these strains harbored a variety of virulence genes that could cause multiple diseases, thereby posing a serious threat to food handlers and consumers. Both MRSA and MSSA isolates showed multidrug resistance; in particular, the MRSA-III strain exhibited significant antimicrobial resistance. Identification of STs showed that the genetic diversity of *S.aureus* in aquatic food was high and that different STs were associated with specific virulence genes and antimicrobial resistance. Thus, our findings provided important insights into the dissemination of these strains.

## Ethics Statement

All procedures performed in studies involving human participants were in accordance with the ethical standards.

## Author Contributions

Conceived and designed the experiments: DR, JZ, MX, and QW. Performed the experiments: DR and SY. Analyzed the data: DR and JZ. Contributed reagents/materials/analysis tools: DR, QW, and JZ.

## Conflict of Interest Statement

The authors declare that the research was conducted in the absence of any commercial or financial relationships that could be construed as a potential conflict of interest.

## Footnotes

1 http://pubmlst.org/databases.shtml

2 http://www.nhfpc.gov.cn/yzygj/s3593/201608/f1ed26a0c8774e1c8fc89dd481ec84d7.shtml
