# Supplementary material for: Prevalence, Virulence Genes, Antimicrobial Susceptibility, and Genetic Diversity of Staphylococcus aureus from Retail Aquatic Products in China
Source: Front Microbiol. 2017 Apr 20;8:714. doi: 10.3389/fmicb.2017.00714 (PMC5398012; doi:10.3389/fmicb.2017.00714)
Supplement: Supplementary file 1 [file Table_1.docx]

Supplementary Material

**Prevalence, virulence genes, antimicrobial susceptibility, and genetic diversity of** ***Staphylococcus aureus* from aquatic products from retail markets in China**

Dongli Rong^1,2^, Qingping Wu^1^, Mingfang Xu^2^*, Jumei Zhang^1^*,Shubo Yu^1^

*** Correspondence:** Mingfang Xu: E-mail: [xmf20142014@163.com](mailto:xmf20142014@163.com)

Jumei Zhang: E-mail: Zhangjm926@126.com

## Supplementary Figures

| Table 1. Results of antibiotic susceptibility and virulence factors corresponding  to ST types of *S. aureus* in retail aquatic products in China | | | | | | |
| --- | --- | --- | --- | --- | --- | --- |
| Strain Number | Citys | Antimicrobial resisitance profiles | Virulence genes | ST types | SCC*mec* | Sources |
| 3381 | Lhasa | / | *sea-see-pvl-hlα-fnbA* | 1036 |  | SF |
| 3377 | Lhasa | P-AMP-AMC-K-E | *sea-see-pvl-hlα-fnbA* | 15 |  | SF |
| 3504 | Hongkong | P-AMP-S-E | *sec-see-hlα-fnbA-fnbB* | 15 |  | SP |
| 3380 | Lhasa | P-AMP-AMC-K-E | *sec-see* | 15 |  | FF |
| 2955 | Changchun | P-AMP-AMC-S-E-SXT | *see-hlα-fnbA* | 15 |  | FF |
| 3479 | Macou | P-AMP-AMC-CN-TE-FD | *sea-sec-see-pvl-hlα-fnbA* | 15 |  | FF |
| 3126 | Huhhot | AMP-AMC-CAZ-E | *sea-seb-sec-pvl-hlα-hlβ-fnbA* | 15 |  | SF |
| 1980 | Harbin | P-AMP-FD | *sec-sed-see-pvl-hlα-fnbA* | 15 |  | FF |
| 2529 | Nanchang | P-AMP-AMC-E | *sec* | 15 |  | FF |
| 3379 | Lhasa | P-AMP-AMC-S-E | *see-pvl-fnbA* | 15 |  | FF |
| 3155 | Shenyang | P-AMP-AMC-K-S-E | *pvl-hlα-fnbA* | 15 |  | SF |
| 3379 | Lhasa | P-AMP-S-E | *sec-hlα* | 15 |  | FF |
| 2531 | Nanchang | P-AMP-FD | *sec-hlα-fnbA* | 15 |  | FF |
| 3156 | Shenyang | P-AMC-E | *sea-see-pvl-hlα-fnbB* | 15 |  | FF |
| 3327 | Zhengzhou | P-AMP-AMC-S-E | *sea-see-fnbB* | 15 |  | SP |
| 3107 | Huhhot | P-AMP-AMC-E | *pvl-hlα-fnbA-fnbB* | 15 |  | FF |
| 2328 | Xiamen | P-AMP-AMC-E | *pvl-hlα-fnbA-fnbB* | 15 |  | SF |
| 2826 | Changsha | P-AMP-AMC-E | *fnbA-fnbB* | 15 |  | SF |
| 2877 | Hangzhou | P-AMP-AMC-E | *pvl-hlα-fnbA* | 15 |  | SP |
| 3157 | Shenyang | P-AMP-AMC-E | *sea-see-pvl-hlα-fnbA* | 15 |  | FF |
| 2606 | Hefei | P-AMP-AMC-E | *sed-see* | 15 |  | SF |
| 2904 | Guiyang | P-AMP-SXT | *see-pvl-hlα-fnbA* | 15 |  | FF |
| 2056 | Beijing | AMP-E | *sec-sed-see-pvl-hlα-fnbA-fnbp fnbB -tiss-1* | 15 |  | FF |
| 3406 | Urumqi | / | *sea-see-pvl-hlα-fnbA* | 15 |  | FF |
| 2807 | Changsha | P-AMP-AMC-S-E | *seb-sec-see* | 1612* |  | SF |
| 3004 | Xining | P-AMP-AMC-NOR-E-TE | *sec-see-pvl-hlα-fnbA* | 959 |  | SP |
| 2780^a^ | Kunming | P-AMP-AMC-FOX-CAZ-FEP-S-E-TEL-C-TE-DA | *see-pvl-hlα-fnbA* | 1 | Ⅲ(3A) | FF |
| 1979 | Harbin | P-AMC-E-DA | *pvl-hlα-fnbA* | 1 |  | FF |
| 2655 | Wuhan | P-AMP-AMC-E-TE-DA | *pvl-hlα-fnbA* | 1 |  | FF |
| 2830 | Changsha | P-AMP-AMC-CIP-E-DA | *sec-see-fnbA* | 1 |  | FF |
| 2657 | Wuhan | AMP-AMC-E | *seb-sec-see-pvl-hlα-fnbA* | 1 |  | FF |
| 3531 | Hongkong | P-AMP-AMC-K-FD | *sea-see-pvl-hlα-fnbA* | 1 |  | FF |
| 2530 | Nanchang | P-AMP-AMC-E-DA-RD | *pvl-hlα* | 1 |  | FF |
| 3430 | Urumqi | P-AMP-E | *pvl-hlα-fnbA* | 1 |  | FF |
| 2779 | Kunming | P-AMP-E | *see-fnbA* | 1 |  | FF |
| 3529 | Hongkong | P-AMP-AMC-K-TE-DA | *sea-see-pvl-hlα-fnbA* | 1 |  | SF |
| 3276 | Shijiazhuang | P-AMP-AMC-CAZ | *see-hlα-fnbA* | 1 |  | SF |
| 2279 | Nanning | P-AMP-AMC | *sea-see-pvl-hlα-fnbA* | 1 |  | FF |
| 2879 | Hangzhou | P-AMP-AMC-TEL | *pvl-hlα-fnbA* | 1 |  | SP |
| 2407 | Haikou | P-AMP-AMC-K-C-TE | *sed-see-hlα-hlβ* | 1 |  | SP |
| 2077 | Beijing | P-AMP-AMC-E | *sec-see-pvl-hlα-fnbA* | 1 |  | SP |
| 2054 | Beijing | P-AMP-AMC-E | *see-hlα-fnbA-fnbB* | 1 |  | FF |
| 2930 | Guiyang | P-AMP-E | *sea-sed-fnbA* | 1 |  | FF |
| 2927 | Guiyang | P-AMP-AMC-E | *see-fnbA* | 1 |  | SP |
| 2778 | Kunming | P-AMP-AMC-FOX-CAZ-FEP-AK-K-S-E-TEL-C-TE-DA | *see-hlα-fnbA-tiss-1* | 1 |  | FF |
| 3128 | Huhhot | P-AMP-AMC | *sec-see-hlα-fnbA* | 1 |  | SP |
| 2676 | Wuhan | P-AMP-AMC | *sed-see-pvl-hlα-fnbA* | 1 |  | SP |
| 2629 | Hefei | P-AMP-AMC | *pvl-hlα-fnbA* | 1 |  | FF |
| 2405 | Haikou | AMP | *sed-see-pvl-hlα-fnbA* | 1 |  | FF |
| 3028 | Xining | P-AMP-S | *sea-see-pvl-hlα-fnbA* | 1 |  | SF |
| 3106 | Huhhot | P-AMP-AMC-K-NOR-E-SXT | *seb-sec-pvl-hlα-fnbA* | 3304* |  | FF |
| 3026 | Xining | P-AMP-AMC-K-S-CIP-E-TEL-C-TE-FOS-RD | *pvl-hlα-fnbA* | 3304* |  | SF |
| 2581^a^ | Chengdu | P-AMP-AMC-FOX-CAZ-E-TE | *sec-sed-pvl-hlα-fnbA* | 25 | Ⅳa(2B) | FF |
| 2406^a^ | Haikou | P-AMC--FOX-CAZ-CN-TE | *sec-pvl-hlα-fnbA* | 25 | Ⅳa(2B) | FF |
| 2280 | Nanning | P-AMP-AMC-E | *seb-sec-see-pvl-hlα-fnbA* | 25 |  | FF |
| 3154 | Shenyang | P-AMP-AMC-NOR-E | *seb-sec-hlα-fnbA-fnbB* | 25 |  | SP |
| 3029 | Xining | P-AMP-AMC-FEP-E-TE | *seb-sec-hlα-hlβ-fnbA* | 188 |  | FF |
| 2626 | Hefei | P-AMP-AMC-E | *see-hlα-fnbA* | 188 |  | SF |
| 2430 | Haikou | P-AMP-AMC-K-E-RD | *sec-see-hlα-fnbA* | 188 |  | FF |
| 3231 | Nanjing | AMP-AMC-TE | *sea-seb-sec-pvl-hlα-fnbA* | 188 |  | SF |
| 2427 | Haikou | P-AMP-TE | *pvl-hlα-fnbA* | 188 |  | FF |
| 2426 | Haikou | P-AMP-TE | *pvl-hlα-fnbA* | 188 |  | FF |
| 2526 | Nanchang | P-AMC-C | *sec-pvl-hlα-fnbA* | 188 |  | FF |
| 3429 | Urumqi | P-AMP-AMC-CAZ-E | *sea-hlα-fnbA* | 188 |  | FF |
| 2628 | Hefei | P-AMP-AMC | *see-hlα-fnbA* | 188 |  | FF |
| 2978 | Changchun | P-AMP-E | *see-hlα-fnbA* | 188 |  | SF |
| 3356 | Lhasa | P-K | *see-hlα-fnbA* | 188 |  | FF |
| 2876 | Hangzhou | P-AMP-AMC | *sea-hlα-fnbA* | 188 |  | SP |
| 2905 | Hangzhou | P-AMP | *sec-sed-hlα-fnbA* | 188 |  | FF |
| 2630 | Hefei | P-AMC-CIP-E-TE-DA | *pvl-hlα-fnbA* | 188 |  | FF |
| 3055 | Yinchuan | P-AMP-AMC-E-TEL | *see-hlα-fnbA* | 188 |  | SP |
| 3357 | Lhasa | / | *seahlα-fnbA* | 188 |  | SF |
| 2206 | Fuzhou | / | *sec-sed-see-pvl-hlα-fnbA* | 188 |  | SF |
| 2277 | Nanning | AMP-AMC-NOR-TE | *sec-sed-see-pvl-hlα-fnbA* | 88 |  | SP |
| 2304 | Xiamen | AMP-AMC-K-E-TE | *see-hlα-fnbA* | 1608* |  | FF |
| 2029 | Jinan | P-AMP-AMC-S-E-SXT | *sec-sed-see-pvl-hlα-fnbA* | 672 |  | FF |
| 2428 | Haikou | P-AMP-AMC-K | *sec-pvl-hlα* | 1609* |  | SF |
| 2280 | Nanning | P-AMP-AMC-CAZ-K-E | *pvl-hlα-fnbA* | 1607* |  | FF |
| 2855 | Hangzhou | P-AMP-AMC-NOR | *sec-see-hlα-fnbA-fnbB* | 8 |  | FF |
| 2804 | Changsha | AMP-AMC-ET | *see-hlα-fnbA* | 6 |  | FF |
| 2806 | Changsha | P-AMP-AMC | *hlα* | 6 |  | FF |
| 2928 | Guiyang | / | *sea-sed-fnbA* | 6 |  | FF |
| 3226 | Nanjing | P-AMP-E | *hlα-hlβ* | 6 |  | SF |
| 2828 | Changsha | AMP-AMC | *hlα-fnbA* | 6 |  | FF |
| 3080 | Yinchuan | AMP-AMC-E-TEL | *sea-fnbA* | 6 |  | FF |
| 2129 | Taiyuan | P-AMP-AMC-K-TE-DA | *see-pvl-hlα-fnbA* | 7 |  | FF |
| 3206 | Nanjing | P-AMP-AMC-K-CIP-TE-DA | *sea-see-pvl-hlα-fnbA* | 7 |  | FF |
| 1457 | Guangzhou | P-AMP-AMC-K-TE-SXT | *sec-see-pvl-hlα-fnbA* | 7 |  | SP |
| 2705 | Shanghai | P-AMP-AMC-K-TE-SXT | *sed-see-pvl-hlα* | 7 |  | FF |
| 3131 | Huhhot | AMP-CN-K-S-E-TE | *sea-sec-see-pvl-hlα-fnbA* | 7 |  | FF |
| 2576 | Chengdu | P-AMP-AMC-AK-K-QD-E-TE | *sec-fnbA* | 7 |  | FF |
| 2680 | Wuhan | AMP-AMC-TE | *pvl-hlα-fnbA* | 7 |  | FF |
| 3079 | Yinchuan | P-AMP-AMC-K-CIP-TE | *pvl-hlα-fnbA* | 7 |  | FF |
| 2627 | Hefei | P-AMP-K-TE | *hlα-fnbA-fnbB* | 7 |  | FF |
| 2829 | Changsha | P-AMC-E | *seb-sec-see-hlα-fnbA-fnbB* | 7 |  | FF |
| 3207 | Nanjing | P-AMP-AMC-NOR-TE | *sea-see-pvl-hlα-fnbA* | 7 |  | FF |
| 3005 | Xining | P-AMP-AMC-K-QD-TE-DA | *see-hlα-fnbA* | 7 |  | FF |
| 2255 | Nanning | P-AMP-AMC-E | *hlα-fnbA-fnbB* | 7 |  | FF |
| 2926 | Guiyang | SXT-DA | *see-hlα-fnbA* | 7 |  | SP |
| 1456 | Guangzhou | P-AMP-AMC-CN-K-E-C-DA | *pvl-hlα-fnbA* | 72 |  | FF |
| 2778^a^ | Kunming | P-AMP-AMC-K-QD-TE | *pvl-hlα-fnbA* | 398 | Ⅲ(3A) | FF |
| 2831 | Changsha | P-AMP-E | *sea-pvl-hlα-fnbA* | 398 |  | FF |
| 2429 | Haikou | P-AMP-AMC | *pvl-hlα-fnbA* | 398 |  | FF |
| 3077 | Yinchuan | E | *sec-see-hlα-fnbA* | 398 |  | SF |
| 2356 | Beihai | P-AMP-AMC | *pvl-hlα-fnbA-fnbB* | 398 |  | FF |
| 2278 | Nanning | AMP-AMC-NOR-TE | *sea-seb-sec-see-pvl-hlα-fnbB* | 45 |  | SF |
| 2881 | Hangzhou | P-AMP-E | *see-hlα-fnbA-fnbB* | 1640 |  | FF |
| 2906 | Guiyang | P-AMP-E | *pvl-hlα-fnbA* | 3026 |  | SF |
| 2776^a^ | Kunming | P-AMP-AMC-FOX-CAZ-FEP-AK-K-S-E-TEL-C-TE-DA | *see-pvl-hlα-hlβ-fnbA* | 59 | Ⅲ(3A) | FF |
| 2230^a^ | Fuzhou | P-AMP-AMC-FOX-CAZ-FEP-K-S-E-TEL-DA | *seb* | 59 | Ⅳa(2B) | FF |
| 3204 | Nanjing | P-AMP-AMC-K-CIP-TE | *sec-pvl-hlα-hlβ-fnbA* | 59 |  | SP |
| 2404^a^ | Haikou | P-AMP-AMC-FOX-CAZ-FEP-E-SXT | *sec-hlα-fnbA* | 59 | Ⅳa(2B) | SP |
| 2055 | Beijing | P-AMP | *see-pvl-hlα-hlβ-fnbA-tiss-1* | 59 |  | FF |
| 2704^a^ | Shanghai | P-AMP-AMC-FOX-CAZ-FEP-AK-K-S-QD-E-TEL-C-TE-SXT | *seb-hlα* | 338 | Ⅳa(2B) | SF |
| 2754^a^ | Kunming | P-AMP-AMC-FOX-CAZ-FEP-K-S-E-TEL-C-TE-DA | *see-pvl-hlα-hlβ-fnbA* | 338 | Ⅲ(3A) | FF |
| 2581 | Chengdu | P-AMP-AMC-FOX-CAZ-FEP-E-TE | *sec* | 1610* |  | FF |
| 3280 | Shijiazhuang | P-AMP-AMC-CN-K-NOR-DA | *sea-see* | 2196 |  | FF |
| 3528 | Hongkong | P-AMP-AMC | *sec-see-pvl-hlα-fnbA* | 1685* |  | SF |
| 3530 | Hongkong | P-AMP-AMC | *sec-see-pvl-hlα-fnbA* | 1685* |  | SF |
| FF, Freshwater fish; SF, Saltwater fish; SP, Shirmp; * indicates the novel STs; ^a^ indicates MRSA isolates. | | | | | | |
